# Intraoperative Ultrasound Guidance in Breast-Conserving Surgery Improves Cosmetic Outcomes and Patient Satisfaction: Results of a Multicenter Randomized Controlled Trial (COBALT)

**DOI:** 10.1245/s10434-015-4906-4

**Published:** 2015-10-20

**Authors:** Max H. Haloua, José H. Volders, Nicole M. A. Krekel, Alexander M. F. Lopes Cardozo, Wifred K. de Roos, Louise M. de Widt-Levert, Henk van der Veen, Herman Rijna, Elisabeth Bergers, Katarzyna Jóźwiak, Sybren Meijer, M. Petrousjka van den Tol

**Affiliations:** Department of Surgical Oncology, VU University Medical Center, Amsterdam, The Netherlands; Department of Surgery, Medical Center Alkmaar, Alkmaar, The Netherlands; Department of Surgery, Gelderse Vallei Hospital, Ede, The Netherlands; Department of Surgery, Waterland Hospital, Purmerend, The Netherlands; Department of Surgery, Red Cross Hospital, Beverwijk, The Netherlands; Department of Surgery, Kennemergasthuis, Haarlem, The Netherlands; Department of Radiology, VU University Medical Center, Amsterdam, The Netherlands; Department of Epidemiology and Biostatistics, NKI-AVL, Amsterdam, The Netherlands

## Abstract

**Background:**

Ultrasound-guided breast-conserving surgery (USS) results in a significant reduction in both margin involvement and excision volumes (COBALT trial).

**Objective:**

The aim of the present study was to determine whether USS also leads to improvements in cosmetic outcome and patient satisfaction when compared with standard palpation-guided surgery (PGS).

**Methods:**

A total of 134 patients with T1–T2 invasive breast cancer were included in the COBALT trial (NTR2579) and randomized to either USS (65 patients) or PGS (69 patients). Cosmetic outcomes were assessed by a three-member panel using computerized software Breast Cancer Conservative Treatment cosmetic results (BCCT.core) and by patient self-evaluation, including patient satisfaction. Time points for follow-up were 3, 6, and 12 months after surgery. Overall cosmetic outcome and patient satisfaction were scored on a 4-point Likert scale (excellent, good, fair, or poor), and outcomes were analyzed using a multilevel, mixed effect, proportional odds model for ordinal responses.

**Results:**

Ultrasound-guided breast-conserving surgery achieved better cosmetic outcomes, with 20 % excellence overall and only 6 % rated as poor, whereas 14 % of PGS outcomes were rated excellent and 13 % as poor. USS also had consistently lower odds for worse cosmetic outcomes (odds ratio 0.55, *p* = 0.067) than PGS. The chance of having a worse outcome was significantly increased by a larger lumpectomy volume (*p*_trend_ = 0.002); a volume >40 cc showed odds 2.78-fold higher for a worse outcome than a volume ≤40 cc. USS resulted in higher patient satisfaction compared with PGS.

**Conclusion:**

Ultrasound-guided breast-conserving surgery achieved better overall cosmetic outcomes and patient satisfaction than PGS. Lumpectomy volumes >40 cc resulted in significantly worse cosmetic outcomes.

## Breast Cancer

Breast cancer is the most common form of cancer amongst women in Western countries, with over one million cases diagnosed each year. Over recent decades, less invasive surgical techniques such as breast-conserving surgery (BCS) and the sentinel node procedure for axillary nodal staging have been developed with the aim of preserving healthy breast tissue and healthy lymph nodes, reducing morbidity, and improving cosmetic outcome, all without compromising oncological outcomes.^1^–^3^

Nevertheless, BCS results in difficulties in obtaining tumor-free resection margins using a traditional palpation-guided procedure for palpable breast cancer, with margin involvement in up to 41 % of patients. This results in the need for additional treatment such as radiotherapy boost, re-excision, or even mastectomy.^4^–^6^ These additional treatments are psychologically stressful and have a negative impact on the final cosmetic result.^7^–^9^

An important secondary goal of BCS is the achievement of a satisfactory cosmetic outcome, especially since patient survival rates are improving. A number of studies have shown that cosmetic outcomes in BCS have a marked bearing on psychological outcomes. Unfortunately, poor cosmetic outcomes are still observed in up to 40 % of patients.^10^–^14^ Factors influencing cosmetic outcomes include the volume of resected breast tissue,^15^ the site of the tumor,^16^,^17^ postoperative wound complications,^18^ and the amount of radiotherapy, including the radiotherapy boost.^18^–^20^ Cosmetic outcomes following BCS are mainly influenced by excision volumes, with large excision volumes generally resulting in less favorable cosmetic outcomes.^21^

The COBALT trial was designed to address these shortcomings in BCS by comparing ultrasound-guided surgery (USS) with the standard palpation-guided surgery (PGS). The oncological results showed a dramatic reduction in margin involvement, with tumor-involved margins for the invasive component in 3 % of the USS group compared with 17 % in the PGS group. Moreover, optimal excision volume was achieved by using USS, whereas PGS resulted in excision volumes more than twofold too large.^8^ Of the 13,000 patients diagnosed in The Netherlands each year, approximately 9000 patients have palpable breast cancer, of which 75 % can safely undergo BCS with adjuvant radiotherapy treatment.^22^

The improvements attributable to USS may potentially have a positive impact on the final cosmetic outcomes and patient satisfaction. The current analysis aims to assess the cosmetic outcomes and patient satisfaction of USS compared with PGS after 1 year of follow-up.

## Methods

### Trial Design and Patient Population

The COBALT trial was performed in accordance with the Declaration of Helsinki, guidelines for Good Clinical Practice, and the CONSORT statement.^23^ Central and local independent medical Ethics Review Boards of the participating hospitals approved the study protocol (registered at http://www.TrialRegister.nl, number NTR2579).^24^

The COBALT trial was a comparative, two-arm, parallel group, randomized controlled trial (RCT) undertaken in six hospitals in The Netherlands between October 2010 and March 2012. Eligible patients were randomized in a 1:1 ratio to either USS or PGS.^25^ Patients eligible to participate in the COBALT trial were diagnosed with an early stage (T1–T2, N0–N1) palpable invasive breast cancer and were scheduled to undergo BCS. Women with preoperatively diagnosed ductal carcinoma in situ, multifocal disease, a history of neoadjuvant therapy, previous surgical treatment, or radiation therapy of the affected breast were excluded from the study, while patients who had invasive carcinoma with limited or extensive carcinoma in situ diagnosed postoperatively by the pathologist were not excluded.

### Surgical Technique

Although the definition of tumor-free margins used in the current study was ‘no tumor cells at the margin’, the aim of both USS and PGS for all surgeons was to achieve complete tumor removal with surgically feasible healthy tissue margins of up to 1 cm. The closure of the lumpectomy cavity did not involve oncoplastic surgery techniques, thus allowing seroma formation.

### Calculated Resection Ratios

Excess volume resection was defined as a calculated resection ratio (CRR) and calculated by dividing the volume of each specimen by the optimal resection volume, which is the spherical tumor volume plus an arbitrarily chosen, surgically feasible, tumor-free resection margin of 1 cm.^7^,^26^ In an ideal situation, the specimen volume is identical to the optimal resection volume and the CRR equals 1.0.

### Patient Follow-Up

Follow-up was performed at 3, 6, and 12 months after initial surgery and consisted of digital photographs of the breasts and patient self-evaluation. Digital photographs consisted of standardized 4-viewpoint pictures (one frontal, two oblique, and one lateral picture of the breasts, from neck to waist), and were taken during regular outpatient clinic visits using a digital camera of at least 5 megapixel resolution.

### Scoring of Cosmetic Outcome and Patient Self-Evaluation

The COBALT trial used subjective (panel- and self-evaluation) and objective [Breast Cancer Conservative Treatment cosmetic results (BCCT.core) software] cosmetic outcome evaluation techniques. In all these methods, cosmetic outcome of the treated breast was compared with the untreated breast and scored using the 4-point Likert scale, which classifies outcomes as excellent, good, fair, or poor. ‘Excellent’ meant identical to the untreated breast and ‘poor’ indicated a marked difference with the untreated breast.^27^ Patients who underwent mastectomy following primary surgery were scored as ‘poor’ on all three evaluation methods.

#### Panel Evaluation

A three-member panel (one experienced breast surgeon and two laymen) evaluated the pictures. None of the panel members had performed surgery on the patients, and patient information and the study arm were blinded to the observers. The photographs were combined into a PowerPoint^**®**^ presentation of 30–90 slides, with each slide displaying four photographs of a patient at a specific follow-up time point. Breast shape or contour, breast volume, deformity, nipple position, the appearance of the surgical scar, skin alterations, and overall cosmetic outcome were all scored.

#### Computerized Evaluation: BCCT.core

The BCCT.core has been developed in recent years to facilitate cosmetic outcome assessment, improve reproducibility and to allow the comparison of the results from different breast clinics free of subjective individual scores.^28^ The software was used to assess the frontal photographs.

#### Patient Cosmetic Self-Evaluation

Patient self-evaluation was based on a composite questionnaire that included questions on symmetry between the two breasts on different items, including firmness of the breast, nipple position, breast contour, breast volume, appearance of the scar, final overall result, and patient satisfaction with the appearance of the breast.

### Statistical Methods

Patient, tumor, and treatment characteristics of both types of surgery were compared using the Chi squared test, Fisher’s exact test, or the two-independent sample *t* test. Intraclass correlation coefficient (ICC) was calculated to obtain interobserver reliability between the three panel members. The estimated consistency of agreement between the three observers at each follow-up occasion was considered poor when ICC was <0.40, fair when 0.40 ≤ ICC < 0.60, good when 0.60 ≤ ICC < 0.75, and excellent when 0.75 ≤ ICC < 1.00.^29^

All panel members’ scores were combined for each patient separately at a given follow-up time point by finding the mean overall cosmetic outcome score and rounding it to the nearest integer. A multilevel, mixed effect, proportional odds model for ordinal responses was used to model the odds of having a worse cosmetic outcome. Since the outcome was assessed at three different time points by three different methods of evaluation, each patient contributed nine outcome measurements, and the patient was considered as a random effect in the model. Candidate explanatory variables were study center, type of surgery, follow-up occasion, method of evaluation, T-stadium, body mass index (BMI; kg/m^2^), age, re-excision (yes/no), and boost (yes/no). These variables were considered as fixed effects in the model, and only those that were significant were retained in the final model. The effect of specimen volume on cosmetic outcome was investigated separately after excluding women who had undergone mastectomy after the primary surgery. The volume measurements were categorized into two groups (volume of more or less than 40 cc), and were based on the median volume. During revision of pathology reports, four specimens were identified with suspicious differences between volume and weight, and one specimen lacked volume measurement. Volumes of these specimens were therefore imputed using specimen weight (twice measured: in the operation theater and by the pathologist). A separate model was run to investigate patient satisfaction with the appearance of their breast. The proportionality assumptions for all models were checked and fulfilled.

All *p* values were two-sided and a significance level of 5 % was used. All analyses were performed using STATA version 13 (StataCorp LP, College Station, TX, USA).

## Results

### Patient and Tumor Characteristics

A brief overview of patient and tumor characteristics is provided in Table 1. A complete overview of initial oncological results can be found in the study by Krekel et al.^8^ Mean specimen weight, volume, and CRR were significantly smaller in the USS group than in the PGS group (weight: 38 vs. 52 g; volume: 38 vs. 53 cc; and CRR: 1.0 vs. 1.7; all *p* < 0.01). Additional therapy was more frequently required in the PGS group than in the USS group (27 % vs. 11 %, *p* = 0.038) and five patients in the PGS group with tumor-involved margins underwent a mastectomy. Total missing data rates were 14 % (18 patients) at 3 and 6 months, and 12 % (14 patients) at 1 year. One patient refused to participate in follow-up, one patient had major complications due to chemotherapy and could not participate in follow-up, one patient refused follow-up after 3 months, and one patient was excluded from follow-up because she refused radiotherapy. The remaining missing data points per patient were due to the inability to attend the outpatient clinic visit despite several appointments.

**Table 1:** Patient and tumor characteristics

	PGS	USS	*p* Value^a^
Age [mean (95 % CI)]	57.0 (54.6–59.4)	54.4 (51.9–56.8)	0.124
BMI [mean (95 % CI)]	26.6 (25.4–27.8)	26.2 (25.0–27.4)	0.685
Location of carcinoma [*n* (%)]			
Upper outer	48 (70)	31 (48)	0.079
Upper inner	7 (10)	12 (19)	
Lower outer	9 (13)	16 (25)	
Lower inner	5 (7)	5 (8)	
Weight specimen [mean (95 % CI)]	51.9 (43.9–59.9)	37.8 (31.4–44.1)	0.007
Volume specimen [mean (95 % CI)]	53.1 (45.0–61.2)	37.9 (31.2–44.5)	0.004
Diameter tumor [mean (95 % CI)]	2.0 (1.8–2.2)	2.0 (1.8–2.2)	0.983
T-stadium [*n* (%)]			
T1	35 (51)	36 (56)	0.523
T2	34 (49)	28 (44)	
CRR [mean (95 % CI)]	1.7 (1.4–2.0)	1.0 (0.9–1.2)	<0.001
Additional therapy [*n* (%)]			
None	50 (73)	57 (89)	0.038
Re-excision	3 (4)	1 (2)	(0.016 for comparison additional therapy yes/no)
Mastectomy	5 (7)	0 (0)	
Boost	11 (16)	6 (9)	

*PGS* palpation-guided surgery, *USS* ultrasound-guided surgery, *CI* confidence interval, *BMI* body mass index

^a^Chi squared test, Fisher’s exact test, or two independent sample *t*-tests

### Inter-Rater Reliability for Panel Evaluation

The estimated consistency of agreement between the three observers was fair for responses on the first follow-up occasion (ICC 0.59) and good for the next two occasions (ICC 0.69 at 6 months; ICC 0.68 at 12 months).

### Cosmetic Outcome, Including Mastectomies: Ultrasound-Guided Surgery versus Palpation-Guided Surgery

Ultrasound-guided surgery resulted in better cosmetic outcomes than PGS—20 % versus 14 %, respectively, of the overall responses rated excellent. Only 6 % of the responses were poor with USS, compared to 13 % with PGS (Table 2). Overall, a patient had a 45 % reduced chance of a worse cosmetic outcome with USS compared with PGS (odds ratio [OR] 0.55, *p* = 0.067); the odds of having a worse cosmetic outcome became significantly greater by 12 months (OR 2.57, *p* < 0.001). When considering various evaluation methods, the odds of reporting a worse cosmetic outcome were lower for panel- and self-evaluation than for BCCT.core software. Furthermore, the odds of having a worse cosmetic outcome were 2.35-fold higher for T2 tumors than T1 tumors (*p* = 0.010). (Table 3)

**Table 2:** Overall percentages of overall cosmetic outcome and patient satisfaction categories

	PGS (%)	USS
Cosmetic outcome		
Excellent	14	20
Good	51	52
Fair	22	22
Poor	13	6
Patient satisfaction		
Excellent	26	43
Good	54	47
Fair	7	8
Poor	13	2

*PGS* palpation-guided surgery, *USS* ultrasound-guided surgery

**Table 3:** Odds ratios of having worse cosmetic outcome based on the proportional odds model for ordinal responses

	OR (95 % CI)	*p* Value
Excision method		
PGS	1 (Ref)	
USS	0.55 (0.29–1.04)	0.067
Follow-up, months		
3	1 (Ref)	
6	1.21 (0.88–1.66)	0.237
12	2.57 (1.86–3.55)	<0.001
Evaluation method		
BCCT.core	1 (Ref)	
Panel	0.56 (0.41–0.77)	<0.001
Self-evaluation	0.65 (0.47–0.88)	0.006
T-stadium		
T1	1 (Ref)	
T2	2.35 (1.23–4.51)	0.010
BMI	1.08 (1.01–1.16)	0.020

*OR* odds ratio, *CI* confidence interval, *PGS* palpation-guided surgery, *USS* ultrasound-guided surgery, *BCCT.core* Breast Cancer Conservative Treatment cosmetic results, *BMI* body mass index

**Table 4:** Response probabilities of the overall cosmetic outcome at 1-year follow-up after primary surgery

	PGS	USS
BCCT.core		
Excellent	0.04	0.07
Good	0.52	0.63
Fair	0.37	0.26
Poor	0.07	0.04
Panel evaluation		
Excellent	0.07	0.12
Good	0.62	0.69
Fair	0.27	0.17
Poor	0.04	0.02
Self-evaluation		
Excellent	0.06	0.10
Good	0.60	0.68
Fair	0.29	0.19
Poor	0.05	0.03

T-stadium is fixed at the sample proportions of T-stadium II and BMI is fixed at the means of BMI

*BMI* body mass index, *PGS* palpation-guided surgery, *USS* ultrasound-guided surgery, *BCCT.core* Breast Cancer Conservative Treatment cosmetic results

To elaborate on our findings, in Table 4 we report the probabilities of scoring the breast as excellent, good, fair, or poor. For all three evaluation methods, the probability of an excellent or good score was clearly higher with USS than with PGS, and the cumulative probabilities of these responses were 70 versus 56 % for BCCT.core, 81 versus 69 % for panel evaluation, and 78 versus 66 % for patient self-evaluation, respectively.

### Effect of Volume on Cosmetic Outcome, Mastectomies Excluded

The volume measurements were categorized into two groups (volume of more or less than 40 cc). Excision volumes >40 cc resulted in 2.78 higher odds of having worse outcome when compared with volumes ≤40 cc (OR 2.78, *p* = 0.002). When volume was included in the model as a covariate, the difference between the two types of surgery became nonsignificant (OR 1.18, *p* = 0.574). However, of the lumpectomies performed with USS, only 20 % had a volume larger than 40 cc, while the figure for PGS lumpectomies was 56 %. (Table 5)

**Table 5:** Odds ratios of having a worse cosmetic outcome based on the proportional odds model for ordinal responses (women who underwent mastectomy were excluded)

	OR (95 % CI)	*p* Value
Excision method		
PGS	1 (Ref)	
USS	1.18 (0.67–2.07)	0.574
Follow-up, months		
3	1 (Ref)	
6	1.25 (0.90–1.72)	0.182
12	2.66 (1.91–3.70)	<0.001
Evaluation method		
BCCT.core	1 (Ref)	
Panel	0.60 (0.44–0.83)	0.002
Self-evaluation	0.71 (0.52–0.97)	0.033
Volume, cc		
Category 1 (≤40)	1 (Ref)	
Category 2 (>40)	2.78 (1.49–5.18)	0.002^a^
BMI	1.07 (1.01–1.13)	0.018

*OR* odds ratio, *CI* confidence interval, *PGS* palpation-guided surgery, *USS* ultrasound-guided surgery, *BCCT.core* Breast Cancer Conservative Treatment cosmetic results

^a^Linear trend

### Patient Satisfaction

After USS, 90 % of patients were very satisfied or satisfied with the appearance of their breasts compared to 80 % with PGS. The chances of being less satisfied were 86 % lower after USS than after PGS, controlled for follow-up time points, T-stadium, and BMI (OR 0.14, *p* = 0.006). (Table 2)

## Discussion

Oncological outcomes are the primary endpoints of BCS; however, in recent years cosmetic outcome has gained increased interest. The primary results of the COBALT trial showed that USS significantly reduced margin involvement, while the amount of healthy breast tissue excised was smaller. Furthermore, USS resulted in a reduction of additional treatments and healthcare costs.^8^,^30^ The present study showed that USS improves overall cosmetic outcomes at 1 year following surgery. ‘Excellent’ or ‘good’ outcomes were observed in 72 % of patients who had undergone USS, and ‘poor’ cosmetic outcomes were observed in 6 %. Women treated with PGS had a poor cosmetic outcome twice as often as women who underwent USS.

Two studies evaluating cosmetic outcomes following BCS reported ‘excellent’ or ‘good’ scores in 68–93 % of patients.^31^,^32^ These studies were retrospective, in contrast to the present RCT, and used different evaluation methods (i.e. single observer evaluation). The 93 % ‘excellent’ or ‘good’ overall cosmetic outcome score was obtained by patient self-evaluation, the most subjective evaluation method. The scores obtained with the BCCT.core software in these studies were similar to the scores obtained in the present study.

Only one RCT described cosmetic outcomes with patient self-evaluation 3 years after radio-guided seed localization (RSL) or wire localization (WL) for nonpalpable carcinomas. ‘Excellent’ and ‘good’ scores were obtained in 80 % and 76 % of patients, with a ‘fair’ and ‘poor’ cosmetic outcome in 24 % and 19 %, respectively. Excision volumes and reoperations negatively influenced cosmetic results.^33^

In our study, patients were ‘very satisfied or satisfied’ with the appearance of their breast in 90 % of cases after USS and in 80 % after PGS, which is higher than the 83 % described by Losken et al. after oncoplastic surgery, and higher than the 87 % after BCS reported by Eichler et al..^34^,^35^

Different studies have shown that the rate of cosmetic failure is significantly higher if the size of the lump exceeds >50–70 cm^3^, regardless of breast size.^19^,^20^,^36^ In the present study, only 20 % of the volumes were >40 cc in the USS group compared with 56 % in the PGS group (breast size was not evaluated in this study). Other factors influencing cosmetic outcomes included the site of the tumor,^16^,^17^ postoperative wound complications,^18^ and the amount of radiotherapy, including the radiotherapy boost.^18^–^20^ In this study, none of these factors appeared to be of significant influence on the overall cosmetic outcome; complication rates were low but the rate of women receiving boost radiotherapy was high due to national guidelines.

As a consensus on a gold standard for cosmetic outcome assessment is currently lacking and clear guidelines for the assessment of cosmetic outcomes have failed to emerge, current advice is that a range of objective and subjective evaluation methods should be used to evaluate the appearance of the breasts.^13^,^28^,^37^,^38^ Therefore, the present study used three different evaluation methods to obtain an overall cosmetic outcome.

Cosmetic outcome after 1 year seems to be a good predictor of final cosmetic outcome (although there are long-term effects of radiotherapy on breast appearance).^20^,^39^,^40^ Studies have reported that patients with moderate or poor surgical cosmetic outcome (a few months after surgery) have an increased risk of developing breast shrinkage or induration in the long-term, and thereby underline the importance of reducing specimen volumes.^21^,^39^

A poor cosmetic outcome may negatively influence quality of life and could have a marked bearing on the psychological outcome.^10^–^13^ Using an EORTC QLQ-C30 questionnaire, Hau et al. demonstrated that patients with ‘fair’ or ‘poor’ cosmetic outcome after BCS (at both 5 and 10 years follow-up) had significantly worse quality of life scores.^41^ These considerations therefore predict that patients who underwent USS will have a better quality of life than those who underwent PGS; however, long-term follow-up of at least 3 years, including assessment of patient well-being, are expected in the coming years.

## Conclusions

Ultrasound-guided surgery for early-stage invasive breast cancer is superior to PGS as it significantly lowers margin involvement rates, the need for additional therapy, and healthcare costs, and improves overall cosmetic outcome and patient satisfaction. Overall, ‘excellent’ or ‘good’ cosmetic outcomes were reported for USS and PGS in 72 and 65 % of cases, respectively. The improvements in cosmetic results achieved with USS are probably attributable to reductions in both additional therapy and excision volumes.
